# Health systems and policy research evidence in health policy making in Israel: what are researchers’ practices in transferring knowledge to policy makers?

**DOI:** 10.1186/1478-4505-12-67

**Published:** 2014-12-10

**Authors:** Moriah E Ellen, John N Lavis, Assaf Sharon, Joshua Shemer

**Affiliations:** Jerusalem College of Technology, Ha-Va’ad ha-Le’umi St 21, Jerusalem, 93721 Israel; Israeli Center for Technology Assessment in Health Care, Gertner Institute, Chaim Sheba Medical Center, Tel Hashomer, 52621 Israel; Centre for Health Economics and Policy Analysis, McMaster University, 1280 Main Street West, CRL 209, Hamilton, Ontario L8S 4K1 Canada; Department of Clinical Epidemiology and Biostatistics, McMaster University, 1280 Main Street West, CRL 209, Hamilton, ON L8S 4K1 Canada; Department of Political Science, McMaster University, 1280 Main Street West, CRL 209, Hamilton, ON L8S 4K1 Canada; McMaster Health Forum, McMaster University, 1280 Main Street West, Hamilton, ON L8S Canada; Department of Global Health and Population, Harvard School of Public Health, Boston, 677 Huntington Ave, Boston, MA 02115-6018 USA; Sackler School of Medicine, Tel Aviv University, Tel Aviv, 55 Haim Levanon St, Tel Aviv-Yaffo, 6997801 Israel

**Keywords:** Health systems and policy, Knowledge transfer and exchange, Researchers, Survey

## Abstract

**Background:**

Ensuring the use of research evidence in health system management and policy decisions is an important challenge in this century. Knowledge transfer and exchange (KTE) has emerged as a paradigm to address the challenges and start closing the ‘know-do’ gap. This area of work is gaining momentum in most developed countries, yet, to date, no work has been performed in Israel within this area. The purpose of this study was to identify which KTE activities health systems and policy researchers in Israel have undertaken.

**Methods:**

A cross-sectional web-based survey of researchers who have conducted health systems and policy research in Israel was developed. The survey consisted of a demographics section, quantitative scales, and open-ended questions. The survey was sent to all health systems and policy researchers in Israel (n = 125).

**Results:**

The study response rate (28%) was relatively low as compared to other studies in the same field (range of 42% to 88%). Our survey found that more than a third of the health systems and policy researchers in Israel reported that they were frequently or always involved in the following KTE activities: interactions with target audience through the research process (i.e., during developing a research question or executing the research; 35% to 42%) or through formal or informal meetings during conferences, workshops, or conversations (40%). Less than half of the health systems and policy researchers in Israel are engaged in bridging activities aimed to facilitate target audiences to use research.

**Conclusions:**

This is a fairly new area in Israel and therefore the level of engagement of researchers in KTE activities is not very high. The low response rates could be because KTE is a new field in Israel and minimal KTE initiatives have been undertaken. It is preferable to have higher response rates, yet, after several initiatives, this was the outcome. While the findings are relevant, they may not reflect the total population of health system and policy researchers in Israel. Health system and policy researchers in Israel need to be introduced to the benefits and potential advantages of KTE in an organized and systematic way.

## Background

Knowledge derived from research and experience may be of little value unless it is put into practice. Ensuring the use of research evidence in health system management and policy decisions is an important challenge in this century [1]. Knowledge transfer and exchange (KTE) has emerged as a paradigm to address many of the challenges and start closing the ‘know-do’ gap [1]. KTE is defined as “*the synthesis, exchange, and application of knowledge by relevant stakeholders to accelerate the benefits of global and local innovation in strengthening health systems and improving people’s health*” [2]. Health systems research evidence is not always communicated effectively or in a timely manner, and health system managers, policy, and decision makers do not always have the skills, tools, and capacity to find and use research evidence [3, 4].

Numerous barriers and facilitators can influence the use of research evidence to inform decision and policy-making such as i) the local climate and context, ii) poor relationships between researchers, policy makers, and stakeholders, iii) research production that is not timely, relevant, or packaged appropriately, and iv) the role that researchers and/or intermediary organizations play that can facilitate research transfer [2, 4–7]. Within these barriers and facilitators there are two main groups of actors that play a major role: researchers and knowledge users, i.e., managers, decision makers, and policy makers.

A few theories contemplate the disconnect between researchers and policy makers, the most notable being the Two Community Theory [8, 9]. The difference of mentalities, goals, and attitude towards information, language, perceived credibility, and scope of the problem at hand has long been known to cause wariness and sometimes downright rivalry between researchers and policy makers [2, 10–15]. From the researchers’ point of view, a long and tedious study that may have taken years to conduct, falls many a time on deaf ears at worst, or on impatient, get-to the-point ears at best [16, 17]. Even if a researcher managed to find the right policy maker at the right time, which is frequently not the case [5, 18], there are still political forces and agendas to be taken into account, as well as various degrees of bureaucracies [17, 19]. A researcher may be reluctant to present his research to a policy maker due to fear of sacrificing the independence of the research, or due to lack of appropriately trained policy makers using evidence in the first place [20, 21]. From a policy maker’s perspective there are numerous barriers to incorporating the use of research into decision-making such as a lack of personal contact with the researchers and research that is not timely, high quality, or relevant [6, 22]. One systematic review found that interactions between researchers and policy makers and timing and timeliness increased the prospects for research use, whereas individuals’ negative attitudes toward research and their lack of skills decreased the prospects for research use [5, 23].

Numerous frameworks have been proposed as ways to bridge the ‘know-do’ gap and utilize researcher evidence. The traditional frameworks view the path of research from creation to utilization as a logical flow [24]. While this is considered rational and mimics decision-making processes, a holistic view of all the factors and elements that can influence and facilitate the use of research in policy making is needed. A comprehensive framework that includes a holistic view of the health system, addresses the barriers discussed in the literature, and recognizes the contextual influence on evidence-informed decision making has been proposed by Lavis et al. [25] and further developed by Ellen et al. [26, 27]. This framework outlines seven main domains that can be addressed to assist in transferring knowledge to action. These seven main domains are i) establishing a climate for research use, i.e., activities undertaken by the organization and/or the health system to establish a climate where research evidence is used in decision making, ii) research production efforts, i.e., activities taken by researchers, funders, and knowledge users to ensure the production of timely and relevant research, iii) ‘push’ efforts, i.e., activities usually undertaken by researchers or intermediaries (i.e., librarians or knowledge brokers) to disseminate research evidence to potential users, iv) ‘facilitating pull’ efforts, i.e., activities that the health system needs to undertake in order to ensure that the appropriate infrastructure is in place for knowledge users to access the necessary research evidence, v) ‘pull’ efforts, i.e., activities by health system decision-makers to enable the appropriate use of research evidence, vi) ‘linkage and exchange’ efforts, i.e., activities that focus on facilitating relationships between researchers and knowledge users, and vii) evaluation efforts, i.e., evaluations of KTE interventions and outcomes [25, 26]. While frameworks have been proposed to explain the role of research in policy making, empirical evidence to support such ideas or testing of the frameworks is difficult to find [28–30]. The evidence that does exist pertains to individual initiatives (not to a whole framework or process per se) and are usually based on case studies or interview studies [31, 32], and therefore further work is required in this area.

Previous studies have examined the experiences of researchers in influencing health policy. For instance, Lavis et al. [33] surveyed researchers in 10 low- and middle-income countries and found that less than 50% reported that they engaged in KTE activities such as providing systematic reviews of the research literature to their target audience or establishing and maintaining long-term partnerships with their target audience. Another study by El-Jardali et al. [19] surveyed researchers, policy makers, and stakeholders from Mediterranean countries about their views on the health policy making process and involvement in KTE activities. They found that KTE activities were not frequently undertaken by policy makers and researchers in these countries, that research evidence about high priority policy issues was rarely made available, and that the interaction between policy makers and researchers was limited and mostly informal [19].

While researchers have a large role to play in the evidence-informed health policy process, researchers’ experiences with influencing health services managers’ decisions and the policy making process in Israel has not yet been explored. Numerous studies and frameworks exist that explore the influences on evidence informed decision and policy making, and it is clear that context and climate play an important role [4, 25–27]. The context and the application of knowledge to the local context are essential [29, 34, 35]. The use of research evidence and initiatives undertaken to implement KTE cannot be separated from its social context [24, 29]. The political context has been identified as an influential component in determining the importance and use of knowledge in policy [36]. The context can refer to the broad range of characteristics, circumstances, and conditions surrounding the use of research in management and policy making [37]. Each country has its own political context and structural systems that are unique and specific to that country and region. A country’s context can strongly influence the uptake of research evidence in management and policy making, and, therefore, while one can learn from other countries, each country should attempt to understand the current situation in their own context. Thus, while similar studies have been conducted in other countries, only some of the findings can be generalized yet others will be unique to each context. We are not aware of any survey that was conducted in Israel that focused on researchers’ KTE activities. Therefore, the purpose of this study is to explore the experiences of health systems and policy researchers in Israel with KTE.

## Methods

### Developing the survey

A cross-sectional web-based survey of researchers who have conducted health systems and policy research in Israel was developed. The survey consisted of a demographics section, quantitative scales, and open-ended questions. The survey was based on several sources and previous surveys and focused on linkage and exchange activities between researchers and decision makers/policy makers and activities undertaken by researchers to transfer their research to decision makers and policy makers [19, 33, 38]. The original survey was developed and tested in a range of low- and middle-income countries and demonstrated high internal consistency, face, and content validity [38]. More specifically, the survey focused on three broad domains, namely i) push efforts by research producers (i.e., what is transferred to potential knowledge users, with what investments, and with what passive and active strategies), ii) facilitating user-pull efforts (i.e., what is implemented to enable potential knowledge users to access the knowledge as well as building their capacity), and iii) linkage and exchange efforts (i.e., the inclusion of potential knowledge users in the research and KTE process) [38]. Open-ended questions focused on the researchers’ suggestions to improve the role of researchers in facilitating the use of health systems and policy research in health policy making in Israel and asking the participants to provide examples of policies that did or did not make use of the best available research at the time of the policy decision. Whenever possible, the original wording from the previous surveys was retained; nevertheless, some questions were customized to fit the local context.

The survey was pilot tested with a health systems and policy researcher from the region and was modified as necessary, i.e., the feedback suggested that the survey was too lengthy and time consuming and it was therefore shortened and focused on a limited subset of a larger pool of potential questions. After the first launch of the survey (where only six respondents participated) and feedback on the length of the survey, the survey was further shortened to enable a higher response rate. Subsections of the longer survey and the open-ended questions were deleted.

Four researchers in Israel were consulted to determine if translation was necessary and the conclusion was that the majority, if not all, health systems and policy researchers in Israel have a good command of the English language and therefore translation is unnecessary. Furthermore, in a similar study that was conducted in 12 countries in the Eastern Mediterranean Region, the survey was translated to Arabic; however, only 3 respondents out of 238 invitees answered the survey in Arabic [19] and therefore it was decided that this survey did not need to be translated.

SurveyMonkey software was used to design the survey and collect responses [39]. The survey was web-based and no identifiers were associated with the data. SurveyMonkey does not ask respondents to supply any identifying information and therefore the responses were anonymous.

### Selecting the sample

In determining the size of the potential sample, the first author first examined the number of Universities that offered programs in health policy, systems, epidemiology, and public health and the number of faculty in each department, as well as the number of staff located in the research institutes. These two settings were explored first since the majority of health systems and policy researchers in Israel are affiliated either with a University or with a research institute. The first author then consulted with the Chief Research Scientist at the Ministry of Health and the past Director General of the Ministry of Health to determine a potential sample size. Considering the size of the country, the size of this field of research in Israel, the information obtained by the first author regarding University and research institute affiliations, and the tacit knowledge provided by the two key figures in health policy in Israel, it was estimated that there were approximately 100 health systems and policy researchers in Israel. Therefore, due to the manageable size of potential respondents, it was decided that all potential respondents would be invited to participate in the survey. The target was health systems and policy researchers from academic institutions, hospital settings, government agencies, the four health insurance funds, and research institutes. The National Institute for Health Policy was contacted to determine if they had an inventory of health systems and policy researchers in the country and if they were willing to share the contact information with the research team; however, due to privacy concerns, they were unable to provide us with the necessary information.

Therefore, considering that no publicly available database of health systems and policy researchers exists in Israel, we developed our own database. All potential respondents were identified by i) examining publicly available web sites associated with each academic institution (specifically departments or faculties that focused on health policy type research, i.e., schools of public health, epidemiology, health policy and systems), teaching hospital setting, government agencies, the four health insurance funds, and research institutes that focus on health policy in Israel, and identifying those researchers that focused on health systems and policy; and ii) examining the list of research projects that were funded by the National Institute for Health Policy and obtaining the emails of the associated researchers. Finally, the snowball sampling technique was used by adding a question to the survey asking respondents to identify any additional researchers they think should be contacted that might be interested and may have relevant information for our survey; however, no additional names were provided through this method. The sampling frame was purposefully broad and over-inclusive to ensure that as many potential respondents as possible were identified. Furthermore, in order to make clear to potential respondents the specific type of research in which we were interested, we defined health systems and policy research at the start of the survey, i.e., research related to ‘governance, financial and delivery arrangements for health care and population health services. We asked respondents to only answer all subsequent questions with this type of research in mind even if this research constitutes only a small proportion of the research with which they are involved.

### Recruiting the sample

An initial email was sent out, with a link to the survey, from the lead author to all potential respondents. Two weeks after the initial email, the National Institute for Health Policy held their yearly conference which is attended by health systems and policy researchers in Israel. Permission was obtained to set up a table with the survey on the first day of the conference. One month after the conference, a reminder email was sent out by the lead author. One month later, an email was sent by the past Director General from the Ministry of Health asking potential respondents to complete the survey, the rationale being that if a more senior official that is familiar with most of the potential respondents sends out the survey invite, then there will be a positive effect on the response rate.

### Anticipated response rates

Considering that this was the first survey conducted in Israel that focused on KTE, we wanted to anticipate what the potential response rate would be for the survey. We did this by identifying the response rates in survey studies that have been conducted on researchers in the area of KTE, not limited only to health. Studies were identified by i) reviewing all studies that were included in a recent BRIDGE systematic review that examined the factors that increase, decrease, or have no effect on the use of information in policy making [3], and ii) including studies that members of the team have been involved in that were not captured in the BRIDGE systematic review (because the results were only available after the completion of the systematic review). A total of 119 articles were included in the BRIDGE study, and an additional three were included based on study members’ involvement. Of the 122 articles reviewed, 7 included surveys with researchers in the area of KTE, yet of the 7, 2 referred to the same study and therefore a total of 5 studies conducted surveys in this area. Table 1 identifies the study by authors, objective, and country in which the data is related to; as can be seen, response rates varied from 42% to 88%.

**Table 1:** **Response rates of similar studies conducted on researchers in the area of KTE**

Author	Objective	Country	Population surveyed	Response rate out of researchers only
Woodward, Feldman & Snider [40]	To examine the evidence that decision makers receive, how this influences decision making, and to understand how researchers disseminate the information that they generate	Canada	Health care decision makers; health care researchers from five Ontario universities with medical schools and from Ontario’s two northern universities	74% of health care researchers
Landry, Amara, & Lamari [41, 42]	To examine knowledge utilization measurement and provide explanations for knowledge use in the social sciences	Canada	Faculty members of 55 Canadian Universities in the following departments: anthropology, economics, industrial relations, political science, social work, and sociology	42% of academic researchers
Lavis et al. [43]	To explore the use of health services research in public policy making	Canada	Policy makers and researchers (directors of research units funded by two health departments)	88% of research unit directors
Lavis et al. [33]; Cameron et al. [38]	To explore the involvement of researchers in efforts to collaborate with policy makers and providers to bridge the gaps between research, policy, and practice in 10 low- and middle-income countries	Ten low- and middle-income countries: China, Ghana, India, Iran, Kazakhstan, Laos, Mexico, Pakistan, Senegal, and Tanzania	Researchers were defined as individuals who spent at least 10% of their time doing research, which includes the production, synthesis, and sharing of research	64% of researchers
El-Jardali et al. [19]	To explore researchers’ views and experiences regarding the role of health systems and policy research evidence in health policy making in eastern Mediterranean countries	Eleven eastern Mediterranean countries: Algeria, Bahrain, Egypt, Iran, Jordan, Lebanon, Oman, Sudan, Syria, Tunisia, and Yemen	Senior policy makers at national level and senior health systems researchers within national research institutions, universities, and national governments	56% of researchers

Therefore, based on the response rates seen in previous similar studies, the shortened version of the survey, the option to respond to the survey online or in hard copy, and the three reminders, we expected a relatively high response rate.

### Data analysis

All the quantitative responses were exported from the web-based survey to the Statistical Package for Social Sciences (SPSS) and analyzed using descriptive statistics. For close-end questions about frequency of engagement in bridging activities we combined together the two lowest categories (never or rarely engaged) as well as the top two categories (engaged frequently or always) for analysis.

### Ethics

Ethics approval was received from the Jerusalem College of Technology’s (the first author’s primary affiliation) sub-committee on ethics.

## Results

A total of 125 surveys were sent out; 5 were incorrect email addresses, 27 responded to the web-based version, and 5 responded with the hard copy version at the conference, for a total of 32 responses. Considering that we wanted to ensure inclusion of all possible researchers that conducted health systems and policy research, the initial list that was compiled was all inclusive. After further review and consultation with other researchers in the field regarding the list of potential respondents, 13 potential respondents were identified that were not relevant for the study. Therefore, after all things considered, the response rate was 28%. Five of the respondents did not fully answer the survey; however, since we analyzed the data descriptively, per item, we decided not to remove them from the analysis.

In light of the low response rate in our study a non-response bias analysis was considered. The only potential background variable we had for both respondents and non-respondents was place of work affiliation; originally added to the population frame we developed for this study. While asking respondents about their work affiliation, at least half of them mentioned more than one place of affiliation. This finding indicates that the initial information we had about this variable underestimated its true distribution in the relevant population. This fact prevented us from using this variable to compare respondents to non-respondents.

### Demographics

Overall, 55% of the surveyed researchers were males. The mean age was 53 (s.d. = 11.8) and 35% of the surveyed researchers were medical doctors, 15% were nurses, and 60% had a PhD degree; 40% of respondents reported working at universities or academic colleges, 30% at research institutes, 20% at health insurance funds, and 20% at governmental departments (total is more than 100% since some respondents had identified more than one affiliation).

The surveyed researchers were asked about their main domain of health systems and policy research; 15% reported that the main domain of their research was health economics and policy, 15% focused on health information and technology, and about 10% focused mainly on public health topics. The rest of the respondents reported conducting research on diverse topics including epidemiology, trauma, health promotion, and health human resources; 15% of respondents did not specify their main domain of research.

### Engaging in KTE activities

Less than a third of the surveyed researchers reported being frequently engaged in activities aimed to transfer material on health systems and policy research to the target audience (Table 2). The activities that were most often engaged in were developing messages for policy makers or decision makers that specified possible action (30%); providing syntheses of the research literature (29%); and developing brief summaries of articles and/or research reports (25%). Less than 10% of the researchers frequently provided target audiences formal systematic reviews of the research literature.

**Table 2:** **What is transferred frequently or always from researchers to target audiences?**

Activity	Percentage frequently or always
Developed messages for policy makers and/or decision makers that specified possible action (i.e., recommendations, take-home messages, actionable messages)	30
Provided syntheses of the research literature to policy makers and/or decision makers (not including formal systematic reviews of the research literature that follow explicit rules to reduce bias in searching the literature, identifying eligible articles)	29
Developed brief summaries of articles and/or research reports for policy makers and/or decision makers (not including brief summaries of syntheses and/or formal systematic reviews)	25
Provided reprints/copies of articles published in scientific journals to policy makers and/or decision makers (not including syntheses or formal systematic reviews of the research literature)	21
Developed brief summaries of syntheses and/or formal systematic reviews of the research literature for policy makers and/or decision makers	21
Provided formal systematic reviews of the research literature to policy makers and/or decision makers	8

About 40% of health systems and policy researchers reported that they are frequently engaged in supporting activities such as tailoring the content of mailings or e-mails to specific policy makers (Table 3). However, only 10% of the respondents reported being engaged in activities aimed to support their efforts to push health systems and policy research to target audiences such as participating in KTE skill-building activities or working with a KTE specialist or knowledge broker in or outside their organizations. In addition, about 60% of the researchers rarely sent mail or e-mails to a specific target audience that included articles, reports, syntheses, formal systematic reviews, and/or a newsletter containing brief summaries and/or messages (results not shown).

**Table 3:** **To whom, by whom, and how is research being transferred to frequently or always?**

Activity	Percentage frequently or always
Tailored other aspects of your KTE approach to specific policy makers and/or decision makers	41
Tailored the content of mailings or e-mails to specific policy makers and/or decision makers	36
Reviewed the research literature about effective approaches to KTE	24
Developed reports, summaries, or messages that used language appropriate to specific policy makers and/or decision makers (e.g., non-technical, jargon free language)	23
Mailed or e-mailed to policy makers and/or decision makers articles, reports, syntheses, formal systematic reviews, and/or messages without an explicit request	19
Mailed or e-mailed to policy makers and/or decision makers a newsletter containing brief summaries and/or messages	19
Identified and worked with the most credible messengers for policy makers and/or decision makers (i.e., those who, regardless of their role or organization, are seen as credible by members of your target audience)	14
Participated in KTE skill-building activities (e.g., conferences or courses about KTE)	10
Worked with KTE specialists in your organization to promote health systems and policy research	10
Identified and worked with KTE specialists outside your organization	5
Identified and worked with knowledge brokers outside your organization (i.e., “people who bring researchers and their target audiences together and build relationships among them that make KTE more effective”)	5
Developed relationships with print, radio, and/or television journalists	5

About a third of the surveyed researchers frequently engaged in activities aimed to facilitate the target audience’s pull of health systems and policy research or facilitate KTE between researchers and policy/decision makers (Table 4). Respondents reported having involved health services policy and/or decision makers in establishing the overall direction of KTE activities related to the health topic (35%) or in establishing the overall direction of research (26%). Less than a third of respondents provided target audiences with access to a searchable database of brief summaries of articles, reports, syntheses, and/or formal systematic reviews and/or messages that specified possible action (26%). Less than a quarter of the researchers reported being frequently engaged in facilitating activities, such as providing training to target audiences, in order to develop their capacity to acquire, assess, adapt, and apply health systems and policy research (Table 4).

**Table 4:** **What passive strategies and exchange efforts have been used frequently or always to facilitate pull by target audiences?**

Activity	Percentage frequently or always
Involved health services policy makers and/or decision makers in establishing the overall direction of KTE activities related to the health topic undertaken by you and/or your research organization	35
Provided access to a searchable database of brief summaries of articles, reports, syntheses, and/or formal systematic reviews and/or messages that specified possible action for your target audiences	26
Involved health services policy makers and/or decision makers in establishing the overall direction of research on the health topic conducted by you and/or your research organization	26
Provided access to a searchable database of articles, reports, syntheses, and/or formal systematic reviews on the health topic	22
Conducted deliberative dialogues with key stakeholders (dialogues where research evidence can be discussed together with the views, experiences, and tacit knowledge of relevant stakeholders)	22
Provided training to policy makers and/or decision makers to develop their capacity to acquire, assess, adapt, and apply health systems and policy research	17

With respect to engaging in activities that facilitate KTE through interaction with the target audience, it was found that 42% of the surveyed researchers interacted with the target audience when developing a research question, objective, or hypothesis, 35% interacted with the target audience when executing the research or when developing research products, and 40% interacted with the target audience through conferences or workshops or through informal conversations (Table 5).

**Table 5:** **Which linkage and exchange activities are being undertaken frequently or always with particular target audiences?**

Activity	Percentage frequently or always
Interacted when developing a specific research question, objectives, or hypothesis	42
Interacted through conferences and workshops involving your target audiences	40
Interacted through informal conversations with your target audiences	40
Interacted when undertaking KTE activities for your target audiences	39
Interacted when executing the research	35
Interacted when developing research products (e.g., research reports, brief summaries, and/or messages)	35
Interacted through an expert committee or group involving your target audiences	32
Interacted through events organized by you and/or your organization	32
Interacted when analyzing/interpreting the research findings	27
Interacted when establishing the preferred research design and methods	23
Interacted through formal private or public networks involving your target audiences	20
Interacted through government-sponsored meetings involving your target audiences	16
Assessed or participated in assessments of the usefulness and impact of your KTE activities	15

## Discussion

### Summary of study findings

The main purpose of this study was to explore the views and experiences of health systems and policy researchers in Israel with KTE, a subject not yet explored scientifically in Israel. Our survey found that more than a third of respondents reported that they were involved frequently in the following bridging activities: interactions with target audience throughout the research process (i.e., developing a specific research question, executing of the research, or when developing research products; 35% to 42%) or through formal or informal meetings during conferences, workshops, or conversations (40%). Less than half of respondents are engaged in bridging activities aimed to facilitate target audiences’ use of research evidence. In particular, providing access to relevant material about topics of interest and assisting target audiences to develop their capacity to acquire and use research is rarely done.

### Relation to other studies

The response rate in this study (28%) as compared to other studies in the same field is relatively low (range of 42% to 88%). The sampling frame was purposefully broad to ensure that all potential respondents were captured; we recognize that some of the individuals that we included in our list of potential respondents may not have been appropriate candidates, however, they were identified through our targeted search mentioned above and therefore were included. There could be a number of reasons to explain the low response rate. Firstly, for technical reasons, it could be possible that not all potential respondents received copies of the survey as the servers in their institutions may have directed the email from SurveyMonkey to the spam or trash folders. Secondly, it could be that some of the respondents do not conduct or participate in the types of research (i.e., systematic reviews or other syntheses of research findings) that are relevant to policy makers and to support evidence informed decision making [44]. Lastly, considering that KTE is a field that has not been introduced and minimal, if not any, KTE initiatives have been undertaken in Israel, it is possible that there is insufficient interest. In the other countries where similar surveys were conducted, i.e., Canada and Lebanon, KTE initiatives had already been introduced prior to the study and it may therefore have been of higher interest to the potential respondents. While it is preferable to have higher response rates after several initiatives, this was the outcome and, therefore, while the findings are relevant, they may not reflect the total population of health systems and policy researchers in Israel.

These findings coincide with recent studies about KTE that used similar tools and found that engagement in a variety of promising activities are only partially undertaken by researchers [19, 33]. The findings seem to reflect that, in Israel, linkage and exchange activities are undertaken by researchers, i.e., researchers include the target audience in designing the research study, and this is an essential element in ensuring KTE. This seems to be the strongest element within Israel and this can be potentially explained by the fact that Israel is a relatively small country, where the researchers and users are familiar with one another and there are minimal, and in some instances no, barriers to access. However, it seems that in Israel, compared to other countries, activities aimed to facilitate researchers’ engagement in KTE (i.e., KTE skill building or working with KTE specialists) are not common at all. This difference can be explained by the fact that the idea of KTE is rather new in the Israeli health system and researchers are not yet familiar with its potential advantages and contributions to practice.

### Strengths and limitations

Our study has three main strengths: i) the survey itself was built on a pre-existing and validated instrument, ii) numerous initiatives were undertaken to ensure a high response rate (i.e., the survey was computerized and web-based, it was shortened from the original version, and we had an Israeli opinion leader send out an invitation to participate), and iii) this is the first study to explore the practices of health systems and policy researchers on their KTE activities in Israel. However, our survey is not without limitations. The two main limitations are i) the poor response rate in comparison to other surveys on similar populations and ii) the survey is based on self-reports and, therefore, social desirability bias cannot be excluded. These limitations influence the generalizability of the findings to the broader population. Further studies can help to validate and expand on this research.

### Implications and future research

Numerous frameworks, theories, models, and tools have been proposed to explain the role of research in policy making and have highlighted a plethora of activities that researchers can undertake in order to facilitate the use of research in evidence-informed policy making [27–29]. However, as has also been identified in these frameworks, on cannot expect to find investments in all the suggested activities since efforts should be matched to the local context and climate. Therefore, if we use the framework that was originally developed by Lavis et al. [25], where seven main domains are identified, one of the main domains of ‘linkage and exchange’ seems to be somewhat well-established in Israel. Future initiatives for KTE should build on this solid foundation and use these relationships to ensure that the findings from relevant and timely research are disseminated. However, one of the seven main elements is ‘establishing a climate for research use’, and does not strongly exist in Israel. The climate needs to be conducive to linking research to action [25]. To advance this, further work and interventions between researchers and policy makers needs to be developed and implemented to strengthen the culture of the use of research to inform decision making in organizations.

Although our survey was aimed to study researcher’s points of view and experiences with KTE, it should be noted that effective KTE is usually supported by two-way communication. Holmes et al. stressed that researchers, although expected to fulfill certain KTE requirements, should not bear the entire burden of KTE on their shoulders alone [45]. Therefore, while more research needs to be done in Israel on health systems and policy researchers, other actors in the system should also be included to determine their level of interest and potential intervention. For example, policy and decision makers in health insurance funds, and research funders should be surveyed with respect to their perception of the policy making process and potential interventions to increase the use of research in decision making. Furthermore, potential receptiveness to interventions to assist in the use of research to inform decision making should be assessed.

## Conclusions

This research demonstrated that many health systems and policy researchers in Israel are rarely engaged in bridging activities aimed to facilitate target audiences to use research. Providing access to relevant material and assisting target audiences to develop their capacity to acquire and use research is also rarely done. KTE is a fairly new area in Israel and therefore the level of engagement of researchers in KTE activities is not very high. This study is the first stage of a larger proposed program of research that demonstrates the minimal engagement that health systems and policy researchers in Israel have undertaken within a growing field of KTE. Health systems and policy researchers in Israel need to be introduced to the benefits and potential advantages of KTE in an organized and systematic way.

## Abbreviations

KTE: Knowledge transfer and exchange.
